# The Inclusion of a Supercritical Fluid Extract, Obtained From Honey Bee Pollen, in the Diet of Gilthead Sea Bream (*Sparus aurata*), Improves Fish Immune Response by Enhancing Anti-oxidant, and Anti-bacterial Activities

**DOI:** 10.3389/fvets.2020.00095

**Published:** 2020-02-25

**Authors:** Concetta Maria Messina, Valentina Panettieri, Rosaria Arena, Giuseppe Renda, Cristobal Espinosa Ruiz, Maria Morghese, Giovanni Piccolo, Andrea Santulli, Fulvia Bovera

**Affiliations:** ^1^Laboratory of Marine Biochemistry and Ecotoxicology, Department of Earth and Sea Sciences, University of Palermo, Palermo, Italy; ^2^Department of Veterinary Medicine and Animal Production, University of Naples Federico II, Naples, Italy; ^3^Institute of Marine Biology, Consorzio Universitario Della Provincia di Trapani, Trapani, Italy

**Keywords:** honey bee pollen, supercritical fluid extraction, antioxidants, immune-stimulation, *Sparus aurata*

## Abstract

In the present study, the immune-stimulatory effect of two levels of honey bee pollen (5 and 10%, P5 and P10 treatment, respectively) and its supercritical fluid extract (0. 5 and 1%, E0.5 and E1, respectively) included in the diet, was tested in gilthead seabream *(Sparus aurata)*. The *in vivo* trial was preceded by the evaluation of antioxidant properties of three different bee pollen extracts obtained by water, ethanol 80%, and Supercritic Fluids Extraction (SFE). The preliminary evaluation attested that the SFE showed the lowest extraction yield (10.47%) compared to ethanol 80% (48.61%) and water (45.99%). SFE extract showed good antioxidant properties with high polyphenol content (13.06 mg GAE/g), radical scavenging activity (3.12 mg/ml), reducing power (38.68 mg/mL EC50). On the contrary, the water extract showed the significantly lowest polyphenol content (2 mg GAE/g; *P* < 0.05). The results of *in vivo* trial demonstrate that the diets supplemented with SFE bee pollen extract had a stimulatory effect on fish serum immunity, respect to the inclusion of raw pollen, this latter revealing some inhibitory effects in the immune response, such a decrease of serum peroxidase and lysozyme activities, particularly in P10 group significantly different (*P* < 0.05) from the control group. On the contrary, serum peroxidase, protease, antiprotease, were significantly increased in fish fed the diets supplemented with supercritical fluid extract, respect to the fish fed on control and on diets supplemented with 5 and 10% of raw pollen. For what concerns the bactericidal activity against *Vibrio harveyii*, all the treatments containing bee pollen regardless of the type showed their serum bactericidal activity significantly increased with respect to the control groups (*p* < 0.05). Given its high antioxidant properties, the absence of toxic solvents and the positive action carried out on improving the humoral response in gilthead seam bream, honey bee pollen SFE extract can be taken into account in the formulation of fish feeds.

## Introduction

Recently, there has been an increased interest on using plant, medicinal herbs and others compounds as natural immunostimulants, able to enhance the disease resistance in cultured fish (1). Products of vegetable origin seem to represent a very promising source of bioactive molecules, being both easily available, cheap, and biocompatible (2). Some plants, certain parts of them or the extracts, can improve fish health enhancing both the innate and the adaptive immune response, against bacteria, viruses, or parasites (1, 3). This action is represented by the modulation of some enzymes or complexes such as lysozyme, complement, anti-protease, peroxidase, or by the ability to improve some activities as respiratory burst, phagocytosis, antibody production, antioxidant, anti-stress, anti-helminthic, anti-protozoa, anti-fungal, anti-bacterial, and anti-viral (1–4). The use of natural compounds due to their biocide activities represents a promising substitute to the use of the antibiotics and vaccines which are commonly applied in aquaculture to control fish and molluscs diseases (1, 2). In addition, many of these vegetable-derived products have other positive benefits for fish, including enhanced growth, increased weight, appetite stimulation, and they also have the ability to facilitate the sexual maturation of farmed species, while acting as anti-stress and anti-infection agents, including many other health benefits (4).

Honey bee pollen (HBP), in particular, is becoming increasingly popular as functional food for human consumption as a potential source of energy and for its high content of compounds with positive health effects, such as essential amino acids, antioxidants, vitamins, and lipids (5, 6). Pollen preparations are distributed worldwide for dietary scope and as diet integrators. In the USA bee pollen is described by the Dietary Supplement Health and Education Act of 1994 as Dietary Supplement employed to supplement the diet by increasing the total dietary intake (7). The pollen collected by bees contains nutritionally essential components such as carbohydrates, proteins, amino acids, vitamins, mineral substances and trace elements, but also lipids, such as fatty acids, sterols, and several other bioactive compounds (6). Polyphenol substances, mainly flavonoids, are considered, among the ingredients, the principal biomarkers of quality, and may be used for the establishment quality standards for health control of commercially distributed pollen preparations and in relation to their nutritional-physiological properties (7). The bioactive quality of bee pollen decreases over time, and that conditioning carried out on fresh pollen before storage affects its nutritional and functional value (6, 8). In order to extend the *shelf life* of bee pollen and avoid rapid fermentation and deterioration, a dehydration process (artificial drying) is necessary, since its composition has a high level of moisture. In recent years, the growing interest in the extraction and determination of these beneficial bee pollen compounds, has been confirmed by the number of published researches on this topic (6).

For the extraction of natural compounds, solvents with different polarity, from water, to hydro-alcoholic solution are used (9, 10). The antioxidant activity of the extracts and their extraction yield are influenced by the polarity of the solvent. Therefore, the use of different solvents is related to the nature of the polyphenols in the samples (11). For the extraction of many compounds, among these also phenolic ones, the supercritical fluid extraction (SFE) technology is applied. This alternative and green method is often put in comparison to traditional extraction methods with different solvents (ethanol and water), in terms of yield and product quality evaluated by the antioxidant activity of the extracts (10). Today, bee pollen has been used for improving chicken, mammal, and fish growth (12–14). However, there are few studies on the use of bee pollen and its extract to improve welfare and immunity of fish against fish pathogens. Our recent study on meager (*Argyrosomus regius*) demonstrated a negative effect of the addition of raw pollen in the diet on growth, diets digestibility, intestinal traits, and biochemical markers related to health and stress (15), probably due to the structure of bee pollen grains. In view of these results, in the present study on gilthead sea bream, we wanted to verify whether these negative effects could be overcome by the inclusion in the feed of the bioactive fractions previously extracted from pollen, using advanced techniques such as SFE, which does not involve the use of solvents.

Therefore, the aim of the present study was to evaluate the effect of the inclusion of raw and SFE extracted pollen on the immunity response of *Sparus aurata*, one of the most important fish species for Mediterranean aquaculture, so that the results emerging from the trial could potentially found practical applications. A preliminary evaluation of the antioxidants properties of HBP extracts, obtained by chemical and SF extractions, was done. Among immune-related parameters we focused on the activity of different enzymes present in the serum of sea bream (peroxidase, protease, anti- protease, and lysozyme), which are considered as good descriptors for health status in marine organisms (16, 17). In addition, bactericidal activity of *S. aurata* serum against two pathogenic *Vibrio* species were evaluated.

## Materials and Methods

### Extraction With Solvents

About 2 kg of HBP from chestnut, purchased from a local organic farm located in the city of Naples (Napoli, Italy), and collected in about 1 week, were utilized for the trials. For the extractions ethanol 80% and water (1:10 w/v) were used (10, 18). The materials were then homogenized according to a consolidated protocol (19–22). The matrices extracted were centrifuged and then filtered (Whatman® qualitative filter paper, Grade 93–10 μm, Merck KGaA Darmstadt, Germany) and freeze-dried (10, 18).

### Supercritical Fluid Extraction (SFE)

A supercritical extraction unit (SFE System model HELIX, Applied Separations Allentown, PA, USA) was used.

Before the dynamic extraction, a static treatment with SC-CO_2_ was carried out to break the cell walls of bee pollen (5). Dynamic extraction was conducted on dried bee pollen following the method applied by Xu et al. (5) with some modifications. For each extraction the dried powder and hydroscopic dispersing agent (Applied Separations, Allentown, PA, USA) were mixed and placed in the extraction vessel, the unit was pressurized, and dynamic extraction was carried out at pre-established conditions of temperature and pressure with a CO_2_ and a co-solvent flow for 2 h. An additional extraction hour was applied changing the CO_2_ and co-solvent flow.

The obtained extract was stored at −20°C and subsequently extracts were dried.

### Characterization of the Antioxidant Power of HBP Extracted by the Three Different Extraction Methods

#### Total Polyphenols Contents

Total phenolics were analyzed using Folin–Ciocalteu's assay. Gallic acid was used as standard and results were expressed as mg of gallic acid equivalents (GAE) per g of extract of bee pollen (10, 22, 23). Each sample was analyzed in triplicate.

#### DPPH Radical Scavenging Activity

The DPPH (1,1-diphenyl-2-picryhydrazyl) radical scavenging activity was assessed using the method described by Bernatoniene et al. (24) slightly modified by Messina et al. (22): 400 μL of various concentrations of the extracts was replenished up to 2.0 mL with 0.1 mM DPPH radical solution in ethanol.

After 30 min of incubation the absorbance was read against the blank at 517 nm. Gallic acid was employed as the reference. Inhibition of DPPH free radical in percent (I%) was calculated as given below (25):

$$I\%=1-\left(\frac{Asample}{Ablank}\right)*100$$

where Ablank is the absorbance of the control reaction, and Asample is the absorbance of the test sample. The results are expressed as IC 50, mg/mL (26).

#### Reducing Power

The power of the extracts to reduce iron (III) was determined according to the method of Oyaizu (27). Three hundred microliter of sample solutions at different concentrations were mixed with phosphate buffer and potassium ferricyanide 1%; the mixture was incubated at 50°C for 20 min. Three hundred microliter of trichloroacetic acid (10%) was added to the mixture, prior to centrifugation. The upper layer of solution (300 μL) was mixed with distilled water (300 μL) and FeC_13_ (600 μL, 0.1%), and the absorbance was measured at 700 nm against gallic acid as standard. The results are expressed as EC50, mg/ mL (25, 28).

### Fish and Experimental Conditions

The trial was performed in the experimental aquaculture facility of the Department of Veterinary Medicine and Animal Production of Federico II University (Naples, Italy) in respect of the Directive 2010/63/EU and was approved by the University Federico II Ethical Committee and authorized by the Italian Ministry of Health, authorization n. 651/2017-PR; it lasted 30 days and was carried out in an indoor marine water recirculating system (Italian Ministry of Health authorizations n. 78/2013-A and 25/2019-UT) using 90 gilthead sea breams (294.7 ± 12.8 g average initial body weight—IBW) supplied by a local fish farm. The system assured control of water temperature and was equipped with mechanical sand filter, biological filter and UVA sterilization lamp apparatus.

The trial started after a 15 days adaptation period of the fish to the experimental conditions. The water quality parameters were as follows: daily water renewal <1%, artificial day length 12 h, temperature 22 ± 1.5°C, salinity: 33.0 ± 2.0 g/l, dissolved oxygen 6.5± 1.1 mg/l, pH 7.9 ± 0.5, total ammonia nitrogen <0.3 mg/l, nitrite <0.01 mg/l, nitrate <38 mg/l).

On a daily basis, water temperature with a mercury thermometer, pH with an Orion digital pH meter and dissolved oxygen with an oxygen meter (WTW, OXI 330, Weilheim, Germany) were measured daily.

Bi-weekly, total ammonia nitrogen (N-NH3), nitrite-nitrogen (N-NO_2_) and nitrate nitrogen (N-NO_3_) were determined by colorimetric methods, using commercial kits and a spectrophotometer (Hanna Instruments, C-203, Leighton Buzzard, UK).

#### Fish Diets

Fish were randomly distributed in 15 fiberglass 220 l tanks (6 fish per tank) and were fed 5 isoenergetic and isoproteic diets. Each diet was randomly assigned to 3 tanks: a control diet; two diets in which HBP was included at 5% (P5) and at 10% (P10) and two diets in which HBP extract, obtained by SFE, was included at 0,5% (E 0.5) and at 1% (E1). The two pollen inclusion levels were chosen in order to have the same content of total polyphenols both in diets containing raw pollen (P5 and P10) and in diets containing the SFE extracts (E0.5 and E1). The diets were formulated to meet nutrient requirements of gilthead sea bream (29, 30).

The ingredients and chemical composition of the experimental diets are reported in Tables 1, 2, respectively. The diets were physically constituted at the laboratories of the Department of Veterinary Medicine and Animal Production, Napoli Federico II University (Naples, Italy). The HBP was finely chopped, mixed with 10 ml of water to create a paste, then mixed in fish oil and incorporated into the mixture. For E 0.5 and E1 diets, the extract was dissolved initially in 10 ml of fish oil, then incorporated into the mixture. Before the final mixing, all ingredients were ground through a 0.5 mm sieve, then water was added, and the mixture was pelleted through a 3 mm dye using a meat-grinder (Bosh mod. MFW68660, Germany). The diets were then dried in a ventilated oven at a temperature of 40°C for 24 h. This temperature was chosen as it is very similar to the temperature maintained in the hive (34.6°C on average) in order to preserve the pollen quality. The feeds were stored at 4°C until use. Each diet was administered twice a day (09:00 and 16:00 h), 7 days per week. Feeds were administered at 1% of the average body weight. At the end of the trial, all the fish were individually weighted and final weight and FCR were group/tank determined.

**Table 1:** Ingredients of control diets provided to *Sparus aurata* (g/Kg).

**Ingredients**	**Control**
Soy bean meal	240.0
Fish meal	210.0
Corn gluten	190.0
Fish oil	160.0
Gelatinized starch	100.0
Wheat gluten	80.0
Mineral	10.0
Vitamins	10.0

*The experimental diets (P5, P10, E0.5, and E1) were obtained subtracting from the premixed control diet an equivalent quantity to that of the substance to be added (50 and 100 g of honey bee pollen HBP were added to the diets P5 and P10; 5 and 10 g of SFE from HBP were added to the diets E0.5 and E1)*.

**Table 2:** Chemical composition (% as feed) of experimental diets provided to *Sparus aurata* (diets P5 and P10: inclusion of honey bee pollen HBP at 5 and 10%; diets E0.5 and E1: inclusion of SFE from HBP at 0.5 and 1%).

	**Control**	**P5**	**P10**	**E0.5**	**E1**
Dry matter	88.94	88.19	87.63	88.43	88.62
Ash	5.5	4.99	4.35	4.71	4.51
Crude protein	39.78	39.92	38.26	38.91	39.46
Ether extract	17.85	17.25	17.15	17.75	17.15
Crude fibers	7.17	7.54	8.63	7.37	7.62

#### Sampling

After 30 days, on the day of the sampling, three fish per tank (336.2 ± 11.4 g average final body weight—FBW) were euthanized (over anesthesia (MS222-Tricain Metansulphonate at 250 mg/L dosage) and their weight and length were measured. Immediately, blood was collected from the caudal vessel via 5 ml sterile syringes. Blood samples were collected in tubes with separator gel and are left to clot at 4°C for 4 h. Serum were collected after centrifugation (10,000 rpm, 10 min, 4°C) and stored at −80°C.

### Serum Immune Parameters

#### Peroxidase Activity

The peroxidase activity in serum of sea bream fed the different diets, was measured, according to Quade and Roth (31) by oxidation of 3,3′,5,5′-Tetramethylbenzidine (TMB). Briefly, 5 μL of serum were diluted with Hanks's buffer (HBSS) without Ca^+2^ or Mg^+2^ to a final volume of 50 μL in a flat-bottomed 96-well plate. Were added 100 μL of 10 mM TMB with 0.025 of 30% H_2_O_2_, as substrate, and the color change reaction was stopped by adding 50 μL 2 M H_2_SO_4_. The OD was read at 450 nm in a plate reader. Sample without serum were used as blank, and the OD values were subtracted for each sample value. One unit was defined as the amount producing an absorbance change of 1, and the activity expressed as U mL^−1^ for the serum samples. All samples were analyzed in triplicate.

#### Protease Activities

Protease activity was quantified, according to the method described by Ross et al. (32), using the azocasein hydrolysis assay. Ten microliter of serum were incubated with 100 μL of ammonium bicarbonate buffer and 125 μL of 2% azocasein (Sigma Aldrich) in sterile eppendorfs overnight (at RT and in agitation). The reaction was stopped by adding 250 μL of 10% trichloroacetic acid (TCA). The mixtures were centrifuged (6,000 g, 5 min), 100 μL of the supernatants transferred to a flat-bottomed 96-well plate, and 100 μL of 1N NaOH added. The OD was read at 450 nm using a plate reader. For the positive controls (100% of protease activity), the serum, was replaced by trypsin (5 mg mL^−1^, Sigma) whereas by ammonium bicarbonate buffer for the negative controls (0% of protease activity). The activity for each sample was expressed as % protease activity in relation to the controls. All samples were analyzed in triplicate.

#### Antiprotease Activities

Antiprotease activity was determined by the capacity of serum to inhibit trypsin activity (33). Briefly, 10 μL of serum were incubated (10 min, RT) with 10 μL of trypsin solution (5 mg ml^−1^, Sigma) in sterile eppendorfs. After were added 100 μL of ammonium bicarbonate buffer and 125 μL of 2% azocasein (Sigma Aldrich) and the mixtures incubated for 2 h at RT. Following were added 250 μL of 10% trichloroacetic acid (TCA) and the samples incubated for an additional 30 min at RT. The mixtures were then centrifuged (6,000 g, 5 min), 100 μL of the supernatants transferred to a flat-bottomed 96-well plate, and 100 μL of 1N NaOH added. The OD was read at 450 nm using a plate reader (100% trypsin inhibition) was used buffer with no sample or trypsin, and for the negative controls (0% trypsin inhibition) was used a combination of buffer and trypsin solution. The activity for each sample was expressed as % trypsin inhibition in relation to the controls. All samples were analyzed in triplicate.

#### Lysozyme Activity

Lysozyme activity was measured according to the turbidimetric method described by Espinosa Ruiz et al. (34). Twenty-five microliter of serum were placed in a flat-bottomed 96-well plate. To each well, 175 μL of freeze-dried *Micrococcus lysodeikticus* in 10 mM PBS, pH 6.2 (0.3 mg mL^−1^, Sigma, UK) was added as lysozyme substrate. The reduction in absorbance at 450 nm was measured over 15 min at 3 min intervals at RT in a plate reader. One unit of lysozyme activity was defined as a reduction in absorbance of 0.001 min^−1^. The units of lysozyme present in serum were obtained from a standard curve made with hen egg white lysozyme (HEWL, Sigma, UK), and the results were expressed as U mg protein^−1^. All samples were analyzed in triplicate.

#### Bactericidal Activity

Bactericidal activity was determined following the method describe by Espinosa Ruiz et al. (34). Samples of 20 μL of serum were added (in three replicates) to the wells of a flat-bottomed 96-well plate. PBS solution was added to some wells instead of the serum as positive control. Aliquots of 20 Ml of the previously cultured bacteria were added and the plates were incubated for 5 h at 25°C. Then, 25 μL of MTT (1 g L^−1^) were added to each well and the plates were newly incubated again for 10 min at 25°C to allow the formation of formazan. Plates were then centrifuged (4,500 rpm, 10 min), and the precipitates dissolved in 200 μL of DMSO. Then, 100 μL from each well were transferred to another flat-bottom 96-well plate. The absorbance of the dissolved formazan was measured at 570 nm. Bactericidal activity was expressed as percentage of non-viable bacteria, calculated as the difference between absorbance of surviving bacteria compared to the absorbance of bacteria in positive controls (100%).

### Statistical Analysis

Statistical differences among the groups were assessed by one-way ANOVA analyses, followed by the Tukey or Games Howell test depending on the homogeneity of the variables. Normality of the variables was confirmed by the Shapiro–Wilk test and homogeneity of variance by the Levene test. The significance level was 95% in all cases (*P* < 0.05). All the data were analyzed by the computer application SPSS for Windows® (version 20.0, SPSS Inc., Chicago, USA).

## Results and Discussions

### Extraction Yield of HBP

**Figure 1:**
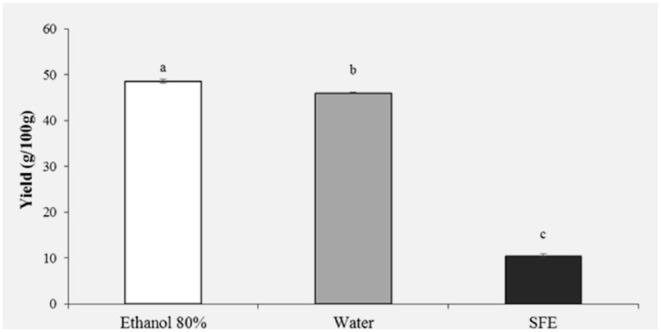
Yield (g/100 g) of honey bee pollen (HBP) extracts obtained with different solvents. Lowercase letters indicate significant differences between different solvents (a, b, c: *P* < 0.05).

The extraction yield obtained from HBP with different solvents (Ethanol 80%, water and SFE) is shown in Figure 1. It was observed that the solvent with the highest extraction efficiency is ethanol 80% (48.61%) followed by water (45.99%). Significantly lower yields were obtained with SFE (10.47%).

The extraction yield and antioxidant activity of plant and other natural extracts strongly depend, both qualitatively and quantitatively, on the polarity of the solvent used during extraction (10, 11).

Previous studies have shown that usually the highest yields are obtained with ethanol, methanol and their mixtures with water (11). Kroyer and Hegedus (7) in their study on bee pollen obtained a yield on aqueous extracts comparable to our results, instead their obtained a lower yield for ethanol extracts.

Total yield obtained from bee pollen using SC-CO_2_ and co-solvent (ethanol 96%) was higher than the one reported by Xu et al. (5), by applying the same pressure and temperature parameters. These authors in fact reach a maximum yield of 5,66% but without using ethanol as co-solvent. Other authors reported that SFE with CO_2_/EtOH could give the best performance, combining extraction yield, and product quality (antioxidant activity and total phenolic compounds) (35).

### Characterization of the Antioxidant Power of HBP

#### Total Polyphenols Contents

**Figure 2:**
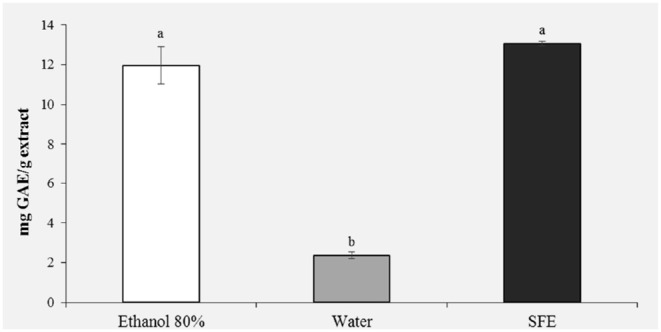
Total phenolic content (mg GAE/g extract) of honey bee pollen (HBP) extracts obtained with different solvents. Lowercase letters indicate significant differences between different solvents (a, b, c: *P* < 0.05).

Figure 2 shows the results of the polyphenol content. It was observed that the highest content of phenolic compounds was recorded in the pollen extract obtained with ethanol 80% (11.96 mg GAE/g) and in the extract obtained with the SFE (13.06 mg GAE/g). With regard to the ethanol extract, this result was expected, being ethanol a very efficient solvent for antioxidant extraction (18). For extracts obtained with SFE, on the other hand, a polyphenol content comparable to the ethanol extract is observed, confirming that extraction with SFE offers greater selectivity, shorter extraction times and does not use toxic organic solvents. It is important to stress how the water extract showed the significantly lowest polyphenol content (2 mg GAE/g; *P* < 0.05).

Given its eco-friendly characteristics and the absence of solvents, extraction with SFE is a suitable technique to produce extracts that can be employed for fish feed.

#### 1-Diphenyl-2-picrylhydrazyl (DPPH) Radical Scavenging Activity

The DPPH test was used to evaluate the scavenging activity of bee pollen against free radicals.

Different values can be found in the literature regarding the activity of scavenging of bee pollen; values between 0 and 97% can be observed in relation to different species of flowers, their chemical composition and extraction with different solvents (36–39).

**Figure 3:**
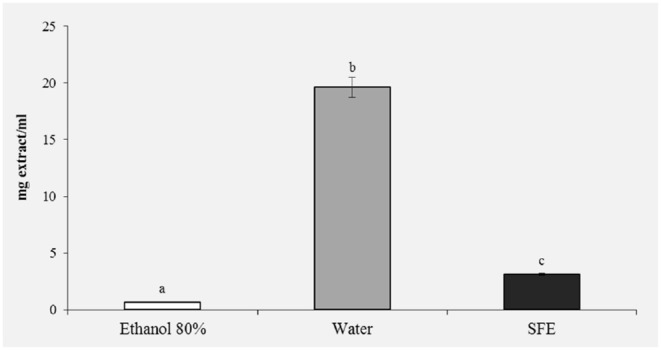
DPPH radical scavenging activity (IC 50, mg/mL) of honey bee pollen (HBP) extracts obtained with different solvents. Lowercase letters indicate significant differences between different solvents (a, b, c: *P* < 0.05).

As shown in Figure 3, ethanol extracts of bee pollen showed high scavenging activity of free radicals with IC50 values of 0.66 mg/ml, similar to those observed by Gabriele et al. (39). Extracts obtained with SFE also showed high scavenging activity (3.12 mg/ml).

#### Reducing Power

The antioxidant activity is improved by the reducing power, which is linked to the presence of reducing agents that, through the donation of a hydrogen atom to free radicals, convert them into stable compounds, breaking the oxidizing chain reaction (10, 40). Through the power reduction assay, the reducing components of a sample can be evaluated directly by measuring the reduction of Fe^3+^ in Fe^2+.^

**Figure 4:**
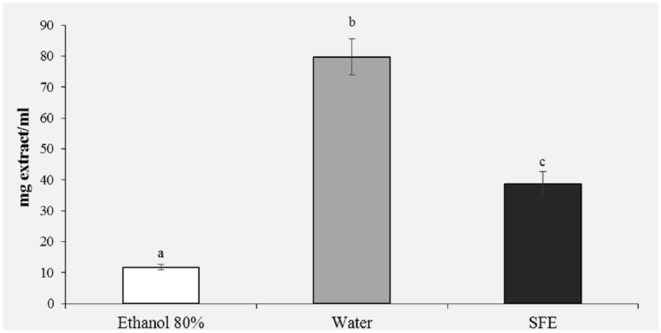
Reducing power (EC50, mg/ml) of honey bee pollen (HBP) extracts obtained with different solvents. Lowercase letters indicate significant differences between different solvents (a, b, c: *P* < 0.05).

In the reducing power assay, the results are expressed as EC50/mg extract, where EC50 value (mg mL^−1^) is the effective concentration of the extract at which the absorbance was 0.5. The results presented in Figure 4 confirm the previous results of the DPPH (Figure 3) and polyphenols (Figure 2); in fact, a greater reducing activity is observed for the samples extracted with ethanol 80% (11.76) and SFE (38.68).

### Serum Immune Parameters Determined in *Sparus aurata*

**Figure 5:**
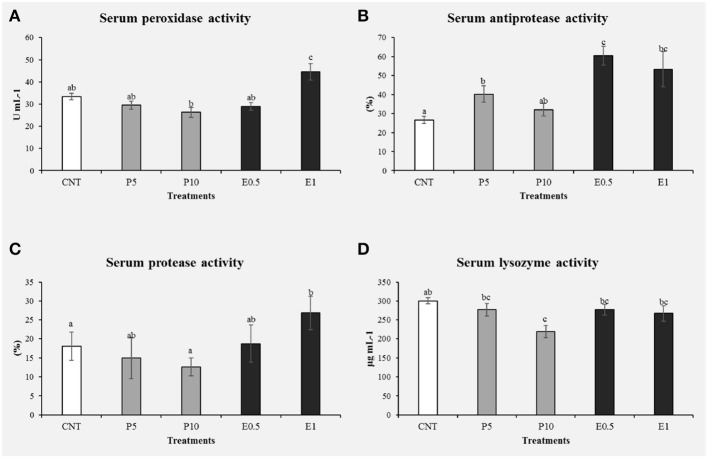
Immune related parameters determined in the serum of *S. aurata* fed with honey bee pollen (HBP) and its SFE extract (CNT: control, P5: pollen 5%, P10: pollen 10%, E0.5: SFE 0.5% and E1: SFE 1%): peroxidase activity (U mg prot−1) **(A)**, antiprotease activity (%) **(B)**, protease activity (%) **(C)**, and lysozyme activity (U mg prot−1) **(D)**. Values are the mean ± SEM (*n* = 9). Statistical differences (*P* < 0.05) between groups are indicated by different letters.

At the end of the feeding period, no statistical differences were found among groups for final weight (336 ± 11.35 g) and FCR (2.3 ± 0.7). Fish fed with both diets P5 and P10 showed some inhibitory effects in the immune response, such a decrease of serum peroxidase (Figure 5A) and lysozyme activities (Figure 5D) respect to the control groups (*p* < 0.05). It is known that the peroxidase enzyme is present in hemocyte granules (41), contributing to the respiratory that involves the production of oxygen metabolites (such as superoxide anion, hydrogen peroxide, and intermediate compounds with high bactericidal activity) (42). After the phagocytosis of microorganisms and other microparticles follows a degranulation process by which these radicals are discharged into the phagosome (43). A reduced peroxidase activity may affect the ability to neutralize pathogens (43), suggesting that the inclusion of 5 and 10% of bee pollen in the diet of sea bream may impair defense capacity. Additionally, lysozyme is a bactericidal enzyme present in the lysosome with an important defense role due to its ability to hydrolyze the components of the bacterial walls (44, 45). The enzyme catalysis the cleavage of β-1-4 bonds between N-acetylglucosamine and N-acetylmuramic acid of bacterial cell wall peptidoglycan, thereby causing bacteriolysis and preventing the growth of bacteria (2, 46). Lysozyme is also known to display anti-viral and anti- inflammatory properties, as well as to activate the complement system and phagocytes by acting like an opsonin (2, 47, 48). During phagocytosis, lysozyme is secreted by hemocytes in the hemolymph, thus inactivating pathogens.

In a recent study, Panettieri et al. (15) highlighted how the use of different inclusion levels of HBP in the diet for meager juveniles led to a worsening of fish growth and nutrients digestibility, along with histological alterations of medium intestine that became more and more severe as the HBP inclusion levels in the diet increased. Such results were supported by immunohistochemistry, hepatic biomolecular markers and blood biochemical analyses. In particular, immunohistochemical detection of TNF-α in medium intestine showed the presence of TNF-α+ cells in the lamina propria and submucosa of bee pollen treated fish, that resulted in accordance with the increased level of the TNF-α protein detected by immunoblotting in the liver. The high hepatic level of HSP70 (*p* < 0.05) in fish fed the diet with the highest inclusion level of HBP (4%) and the linear decrease of total serum protein levels in fish fed the HBP containing diets confirmed the stress situation. The authors attributed these negative effects to the ultrastructure of the bee pollen grains walls that make the bioactive substances unavailable and can irritate the intestine of a carnivorous fish. In particular, the pollen cell walls are composed by stratified concentric layers. The outermost layer is a semi-solid coating composed of neutral lipids, hydrocarbons, terpenoids, and carotenoid pigments. Inside this wall is the exine a matrix of complex carbohydrate, sporopollenin. The exine greatly resists to monogastric digestion and its structure is similar to lignin. These characteristics of raw pollen may make it irritant for the intestinal mucosa and make the bioactive substances at least partially unavailable for carnivorous fish. These aspects could explain the decrease of serum peroxidase and lysozyme activities in P5 and P10 groups compared to the control group.

There are few studies on the use of bee pollen as feed in aquaculture to improve the immunity and protection of fish against fish pathogens. Geay et al. (49) observed that lysozyme levels resulted significantly decreased in *Dicentrarchus labrax* fed on a plant-based diet respect to a diet containing fishmeal. An interesting note is that the present study indicates an improvement in lysozyme levels with the inclusion of HBP extract (0.5 and 1%) in the *S. aurata* diet (Figure 5D). This difference could be related to the total substitution of fishmeal with vegetable proteins in the case of the study by Geay et al. (49), or may indicate that the percentages of pollen inclusion (5 and 10%) used in our study are too high, as they may have interrupted the amino acid profile of diets. Likewise, in the study carried out by Abd-El-Rhman (50) propolis-ethanolic-extract and crude propolis significantly increased the serum lysozyme activity, so it stimulated the immune response in Nile tilapia. The increased lysozyme activity has been reported after supplementing the fish-feed, with non-specific immunostimulants as a mixture of propolis and herba epimedii extracts (51). For this reason, decreased lysozyme activity may suggest immunosuppression in animals, which may lower resistance to pathogenic bacteria (34).

The protease and antiprotease activities were evaluated in serum from sea bream fed the different diets for 1 month (Figures 5B,C). Specimens fed P5, E0.5, and E1 showed significantly increased their antiprotease activities with respect to the control groups (*p* < 0.05). On the other hand, animals fed the E1 showed the protease activity significantly increased with respect to the control groups (*p* < 0.05).

Protease and antiprotease activities activate and improve the development of several immune components such as antimicrobial peptides, complement, and immunoglobulins (52–55). In particular, proteases degrade proteins into either polypeptides or amino acids (56), being principally carried out to reduce the pathogenicity of bacteria and parasites.

Antiproteases, instead, are protease inhibitors that are known to be involved in phagocytosis, coagulation, complement activation and fibrinolysis (55, 56), and moreover, contributes to the innate immunity of animals through its bactericidal and anti-inflammatory properties (57), that restrict the ability of bacteria to invade and grow in fish, by inhibiting their extracellular enzymes (2, 58).

These results indicate that the pollen extract activated the immune system of sea bream, in accordance with previous studies in which the immunostimulant efficacy of plants, certain parts of the same or even their extracts used in feeding tests on fish has been demonstrated (17, 51, 59–61).

#### Bactericidal Activity

Another objective of the present study was to study the effects of the inclusion of pollen in the *S. aurata* diet against two opportunistic pathogenic bacteria, *V. harveyii* and *V. anguillarum*, which were chosen since they are responsible for infections affecting a variety of marine animals, including fish, crustaceans, mollusks, and cetaceans and also humans.

**Figure 6:**
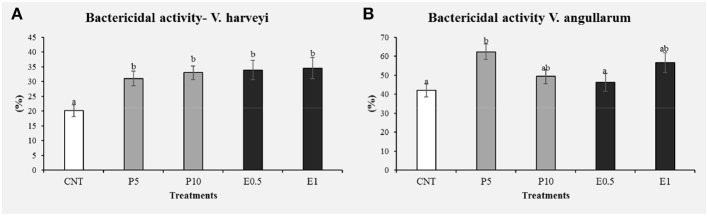
Bactericidal activity against *Vibrio harveyi* **(A)** and *Vibrio anguillarum* **(B)** determined in the serum of *S. aurata* fed with pollen and SFE extract from pollen (CNT: control, P5: pollen 5%, P10: pollen 10%, E0.5: extract 0.5% and E1: extract 1%). Values are the mean ± SEM (*n* = 9). Statistical differences (*P* < 0.05) between groups are indicated by different letters.

Bactericidal activity was evaluated in serum from fish fed the different diets for 1 month (Figures 6A,B). Regarding the bactericidal activity against *V. harveyii*, all the specimens fed the different doses showed their serum bactericidal activity significantly increased with respect to the control groups (*p* < 0.05). On the other hand, only the fish fed with P5 diet showed it bactericidal activity against *V. anguillarum* significantly increased with respect to the control and groups (*p* < 0.05). In addition, Vibrionaceae represents the main cause of mortality in farmed marine species (62, 63). With regard to this, past results show that Gram-positive marine bacteria are usually more susceptible to herbal extracts than Gram-negative marine Vibrionaceae. The results obtained in this study reflect those of similar studies conducted by other authors (14, 50, 64). In previous works honey bee pollen or propolis extracts inclusion in the diet reduced the mortality among *A. hydrophila* challenged fish (50, 64). This benefit is certainly ascribed to the enhanced non-specific immune responses and the antioxidant effects of pollen or propolis extract through its constituent flavonoids, which have antibacterial activity (65).

Therefore, we conclude that pollen SFE at 1% seems to be the best option as supplement in the diet of farmed fish, acting as immunostimulant and antioxidant, able to reinforce the humoral response.

## Conclusions

The supplementation with natural compounds has proved to be a useful tool in aquaculture industry and for this reason, green techniques as SFE, able to reduce the utilization of solvent and to produce extract suitable for nutritional purposes, are encouraged. In the present study, the inclusion of pollen extract in the *S. aurata* diet improved the humoral immunity, as demonstrated by the most common markers related to immunity in fish.

## Data Availability Statement

All datasets generated for this study are included in the article/supplementary files.

## Ethics Statement

The animal study was reviewed and approved by Italian Ministry of Health, authorization n. 651/2017-PR.

## Author Contributions

GP and FB contributed to the conception of the experimental design for the feeding trial with honey bee pollen. CMM and AS contributed to the conception of the experimental design for the alternative methods of extraction, assessment of the antioxidants profile, and immune-related parameters in fish. VP and GP conducted the feeding trials and performed the samples collection. GR and RA performed the extraction by supercritical fluid extraction and the determination of the antioxidants and fatty acids profile of the extracts. CER and MM performed the blood analyses and bactericidal activities and performed the statistical analysis. CMM, GP, AS, and FB supported the acquisition and interpretation of data. VP, RA, MM, CER, and GR drafted the article. CMM, GP, AS, and FB revised and finalized the article. All the authors gave final approval to the manuscript and any revised version submitted.

### Conflict of Interest

The authors declare that the research was conducted in the absence of any commercial or financial relationships that could be construed as a potential conflict of interest.
